# Revealing the clinical impact of MTOR and ARID2 gene mutations on MALT lymphoma of the alimentary canal using targeted sequencing

**DOI:** 10.1186/s13000-024-01525-x

**Published:** 2024-07-25

**Authors:** Xiang Huang, Jiafei Zeng, Yuqing Luo, Shuai Luo, Yao Li, Jinjing Wang

**Affiliations:** 1 Gastroenterology Department, People’s Hospital of Jianhe County, Qiandongnan Prefecture, Guizhou Province P.R. China; 2 Department of Pathology, Affiliated Hospital of Zunyi Medical University, Zunyi, Guizhou Province P.R. China

**Keywords:** Alimentary canal, MALT lymphoma, Next-generation sequencing

## Abstract

Extranodal marginal zone lymphoma of mucosa-associated lymphoid tissue (MALT lymphoma) are a group of diseases with marked heterogeneity, including clinical, immunohistochemical, and molecular heterogeneity. The disease remains unspecified in the genetic landscape with only a few sequencing studies to date; however, systematic studies of alimentary canal MALT lymphoma have not been reported. To better understand the genetics of this tumor, targeted sequencing in a group of 31 cases (including 2 esophageal, 2 colonic, 4 small intestinal, and 23 gastric cases) and two cases of lymph node hyperplasiawere performed. We found epigenetic regulation (*DNMT3A*, *KMT2D*,* KMT2A*,* EP300*,* TET2*, etc.), signaling pathways (*APC*,* CHD8*,* TNFAIP3*,* TNFRSF14*,* ZAP70*,* NF1*,), and tumor suppressor genes (*TP53*,* BCORL1*,* FOXO1*,* ATM*, etc.) involved. Moreover, we found MTOR gene mutations in 16% of the cases that made these patients more prone to recurrence and metastasis than those with *MTOR* wild type genes. More interestingly, *ARID2* mutations were detected in 32% of all the cases, and the mutation rate was higher and statistically significant in *Helicobacter pylori* (Hp)-negative patients in the gastric group. Therefore, this study found that *MTOR* and *ARID2* gene mutations have pathogenic and prognostic implications.

**Supplementary Information:**

The online version contains supplementary material available at 10.1186/s13000-024-01525-x.

## Introduction

Extranodal marginal zone lymphoma of mucosa-associated lymphoid tissue (MALT lymphoma) is the most common form of extradural B-cell lymphoma, accounting for 7-8% of all B-cell lymphomas [1]. The incidence of MALT lymphoma continues to rise with the increase in microbe infections, autoimmune diseases, and secondary immune dysfunctional diseases. MALT lymphoma mainly develops in the gastrointestinal tract accounting for approximately 60–70%, and the esophagus shows low incidence, accounting for < 1% of alimentary canal lymphomas [2, 3]. Alimentary canal MALT lymphoma is a unique type of lymphoma and occurs in different anatomical sites with biological differences, and different genetic variants can lead to varied pathological manifestations, prognosis, and drug resistance [4]. Therefore, we performed targeted sequencing of a group of 31 MALT lymphomas in different parts of the alimentary canal to identify novel genetic mutations that may contribute to tumorigenesis and progression, aiming to better define the genetic landscape of the disease.

## Materials and methods

### Specimen collection

Thirty-one specimens of MALT lymphomas and two lymph node hyperplasia specimens in different parts of the gastrointestinal tract diagnosed and treated at the Affiliated Hospital of Zunyi Medical University from January 2017 to October 2021 were collected, and the proportion of tumor cells estimated using morphology and immunohistochemistry was greater than 75% in all tumor cases. All the cases were diagnosed by hematopathologists in accordance with the World Health Organization (WHO) classification of hematopoietic and lymphoid tissue tumors (revised 4th edition). The experiment was authorized by the Ethics Committee of the Affiliated Hospital of Zunyi Medical University (KLLY-2021-021), and the patients and their families gave their consent.

### Targeted sequencing of selected genes

#### DNA preparation

FFPE tissue samples were dissected into 5 μm thick sections, dewaxed in a xylene bath, washed twice with decreasing percentage of alcohol (100%, 95%, and 70% for 5 min each), followed by washing twice with distilled water for 5 min each. Subsequently, genomic DNA was obtained from the tissue samples using TIANamP FFPE DNA Kit, a Nanodrop 8000 UV-Vis spectrometer (NanoDrop Technologies), a Qubit 2.0 Fluorometer (Life Technologies), and a 4200 TapeStation Instrument (Agilent Technologies, Santa Clara, CA, USA) to verify the DNA concentration and purity.

#### Library preparation and sequencing

Target libraries were designed by using the library building kit named VAHTS^®^ AmpSeqLibrary Prep Kit V3NA210 and the lymphoma-associated 101 gene primer panel, and after checking the library quality, the sequencing template preparation was completed on an Ion Chef™ instrument (Thermo Fisher Scientific) and sequencing was carried out on an Ion GeneStudio™ S5 Plus instrument (Thermo Fisher Scientific) using a 200 bp read length. The panel contains the entire exome of 101 genes associated with lymphoma. Moreover, the raw data were first analyzed using the sequencer server (S5 Torrent Server), then compared to the human reference genome 19 (hg19), and finally, the targeting rate and homogeneity of this sample library this panel were also analyzed. Subsequently, the specific workflow function in Ion Reporter™ Software (Thermo Fisher Scientific) was used to compare our data with hg19 database for mutation calculations, and the analyzed data were reviewed using IGV_2.6.3, after removing the irrelevant data. The resultfor s were left with a sequencing depth greater than 500X and a mutation frequency greater than 5%.The datasets generated and analysed during the current study are available in the [NCBI] repository, [ https://www.ncbi.nlm.nih.gov/sra/PRJNA881268, accession to cite for these SRA data: PRJNA881268 and temporary Submission ID: SUB12058146].

#### Evaluation of the efficacy and prognosis

Patients were evaluated according to their imaging and serological data [5] and were categorized as complete response (CR), partial response (PR), stable disease (SD), and progressive disease (PD). Progressive disease (PD) incidents including recurrence of illness.relapsePatients’ survival was followed up by telephone. Overall survival (OS) was calculated from the time of diagnosis to the time of death or the cut-off time for follow-up.

### Statistical analysis

Statistical analysis was conducted with the help of SPSS22.0 software, and the comparison of rates between groups was made using the fisher’s exact probability method. Furthermore, the analysis of the correlation between mutated genes and prognosis was performed using Spearman correlation analysis.

### Gene Set Enrichment Analysis (GSEA) and Gene Set Variation Analysis (GSVA)

The lymphoma-related data was provided by the TCGA and GEO databases, and the samples were sorted into high and low *MTOR / ARID2* expression groups. The GSVA was performed between both groups using the GSVA package in R.16. Meanwhile, we also performed the GSEA with the datasets described above in order to analyse the difference between high and low MTOR / ARID2 expression groups by means of java GSEA to show the common GSEA plot. Afterward, the commonly activated/suppressed pathways in both groups were identified, and single-gene prognostic analysis was carried out using the R package survimmer.

## Results

**Fig. 1:**
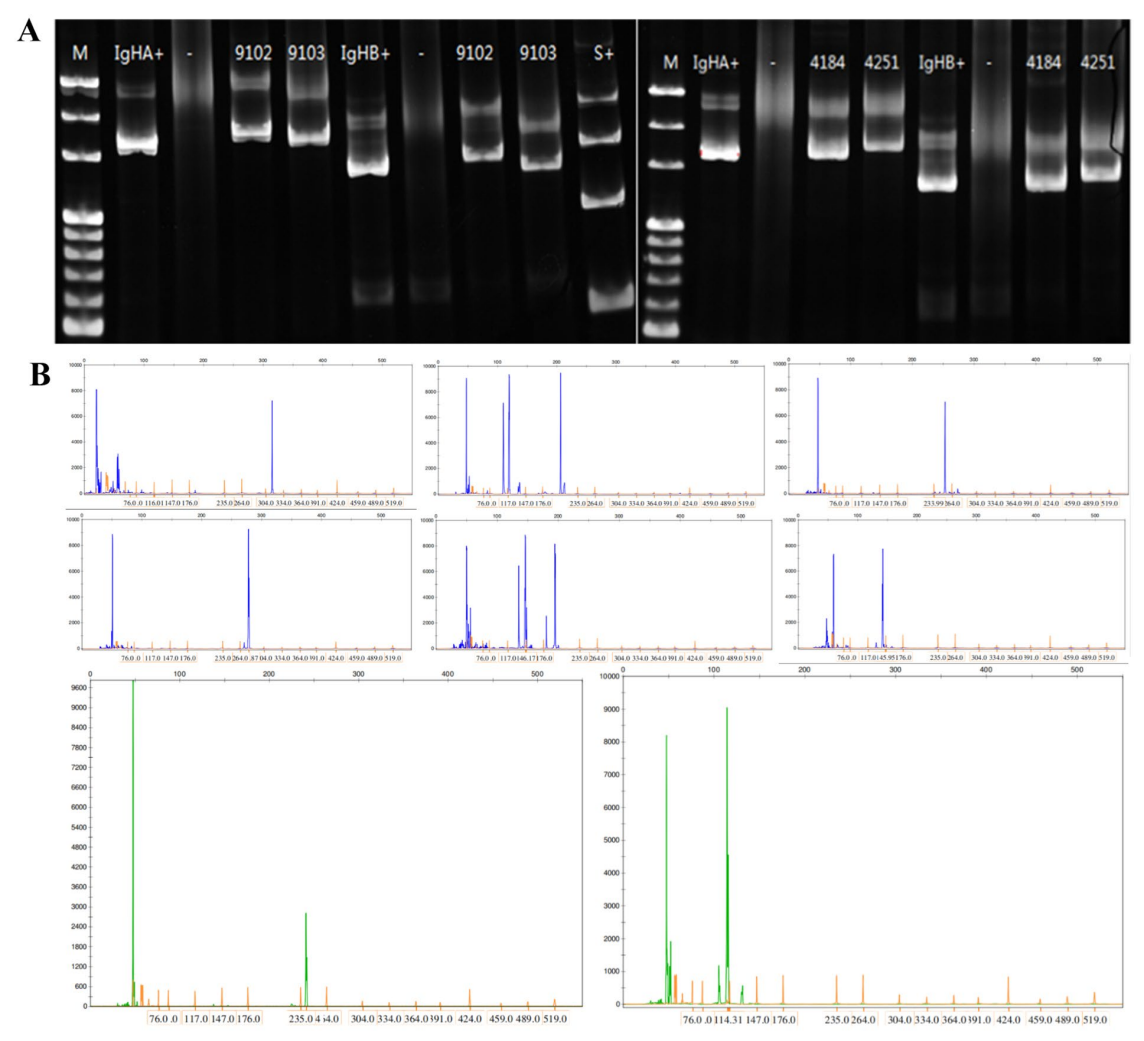
(**A**) Polypropylene gel electrophoresis revealed IGHA, IGHB, IGHC, IGHD, IGKB, IGKA, and IGL with the rearranged bands at 310–360 bp,250–295 bp,100–170 bp,110–290 bp,190–210 bp,270–300 bp and135bp-170 bp, respectively. (**B**) Capillary electrophoresis showed that the IGHA, IGHB, IGHC, IGHD, IGHE, IGKB, IGKA, and IGL peaks were at the corresponding positions, suggesting the rearrangements at the corresponding loci

**Fig. 2:**
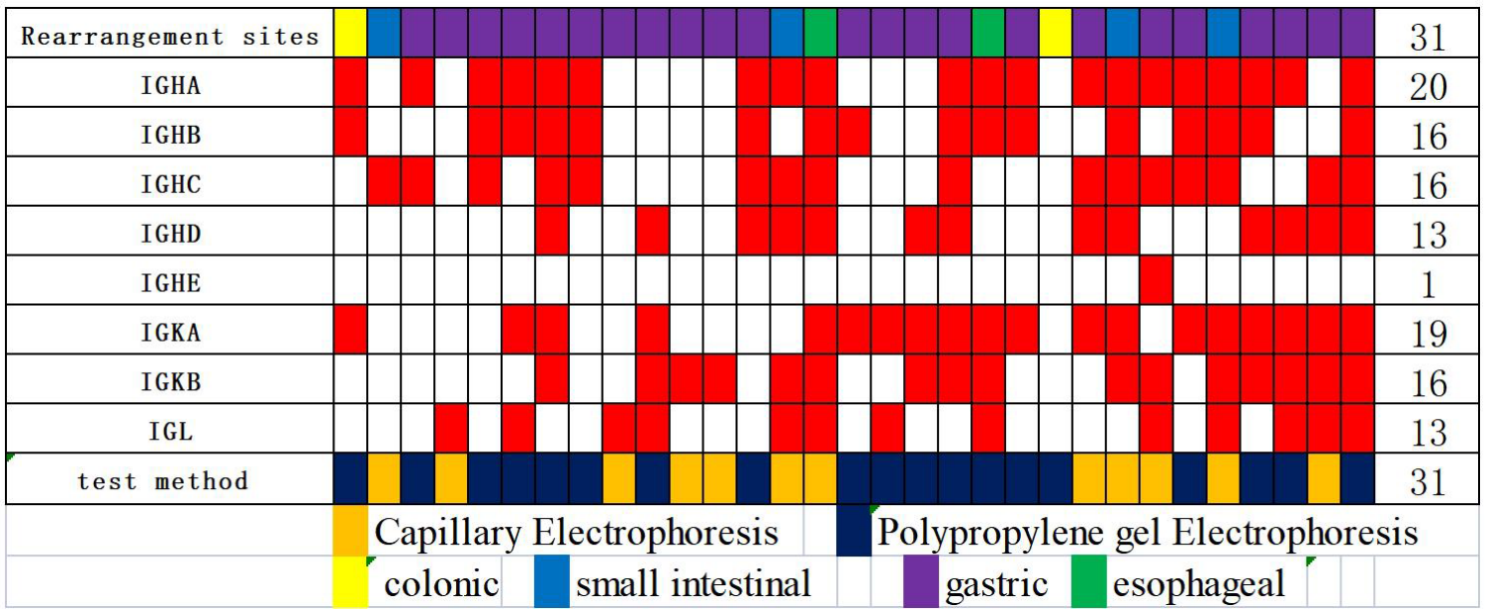
Heatmap of B cell rearrangements of MALT lymphoma of the alimentary canal

**Fig. 3:**
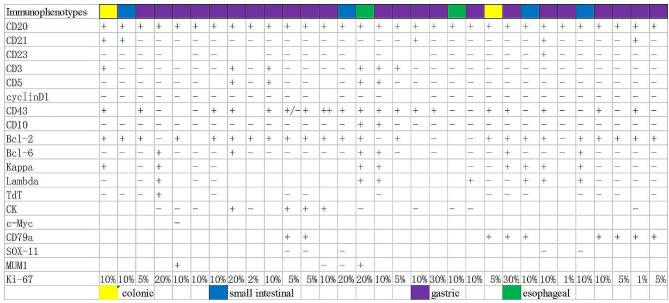
Patient immunohistochemistry results

**Fig. 4:**
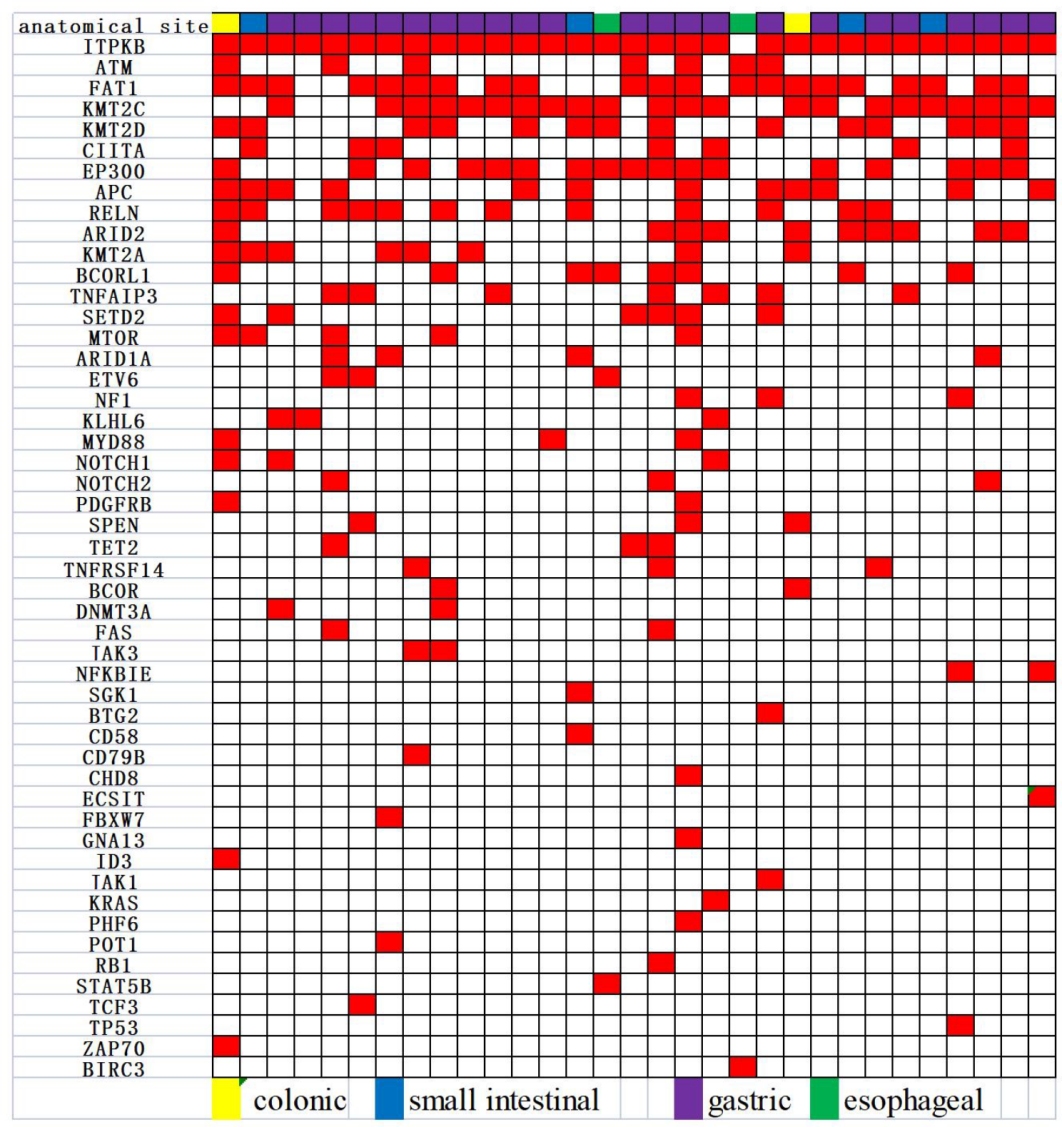
Heatmap of the genetic landscape of MALT lymphoma of the alimentary canal

**Fig. 5:**
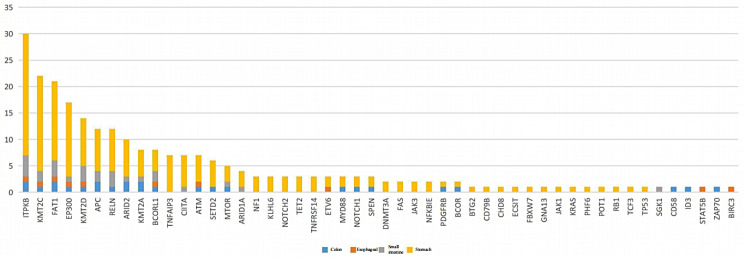
All detected gene mutations, the distribution by site and the total number of mutations

The clinicopathological characteristics of the 31 cases of MALT lymphoma of the alimentary tract are detailed in Table 1. The average age of the onset was 61 years, including 17 males and 14 females. The stomach was the most common site of disease (stomach: 23 cases, esophagus: 2 cases, small intestine: 4 cases, colon: 2 cases). It was observed that 14 cases were positive for *Helicobacter pylori* (Hp) and 9 cases were negative in the stomach group, and 67.7% (21/31) of the patients had clinical stage III/VI. Moreover, genetic rearrangements were detected in IgH, Igκ, or IgL clonal rearrangements (polypropylene gel electrophoresis in 19 cases), Fig. 1A, capillary electrophoresis in 12 cases, Fig. 1B), with IgHA and IgκA loci being the most common (Fig. 2); immunophenotypes: tumor cells showed positive results for CD20, CD79a, BCL-2 were mostly positive, CD3, CD5, CD10, CD23, Cyclin D1, and BCL-6 were mostly negative; CD43 showed the possibility of either being positive or negative, and tumor tissue Kappa or Lambda showed the possibility of restrictive expression (Fig. 3). Gene mutations were detected in 50 genes(Fig. 4), including single nucleotide variants, deletions, or insertions, among which *ITPKB*,* FAT1*,* KMT2C*, and *EP300* had a mutation rate greater than 50% (Fig. 5). *MTOR* and *ARID2* gene mutations were not detected in two cases of lymph node hyperplasia.

**Table 1:** Clinical features of the patients

Variable quantity	Classification	*n*	Percentage(%)
Sex	M	14	45.2%
	F	17	54.8%
Age	<60 years	17	54.8%
	≥ 60years	14	45.2%
Symptom	abdominal pain	23	74.2%
	melena	5	16.1%
	Dysphagia, foreign body sensation	2	6.5%
	abdominal mass	1	3.2%
Stages	I/II	10	32.3%
	III/IV	21	67.7%
IPI grade	0, 1, 2	28	90.3%
	3, 4, 5	3	9.7%
Location	upper gastrointestinal	25	80.6%
	Lower digestive tract	6	19.4%
B symptom	Yes	6	19.4%
	no	25	80.6%
Bone marrow invasion	Yes	4	12.9%
	no	24	77.4%
Lymphocyte count	normal	19	61.3%
	depress	12	38.7%
Albumin	normal	10	32.3%
	depress	21	67.7%
LDH	normal	24	77.4%
	depress	7	22.6%
HP	+	15	48.4%
	-	10	32.3%
Bcl-2	+	23	74.2%
	-	5	16.1%
Bcl-6	+	6	19.4
	-	23	74.2%
Ki−67	≤ 10%	25	80.6%
	>10%	6	19.4%
Endoscopic features	Exelcosis	13	41.9%
	The mucous membrane is white and rough	11	35.5%
Prognosis	CR + PR + SD	28	90.3%
	PD + Death	3	9.7%
Treatment	Eradication of HP, Eradication of HP + chemotherapy	17	54.8%
	Surgery + chemotherapy	14	45.2%

**Fig. 6:**
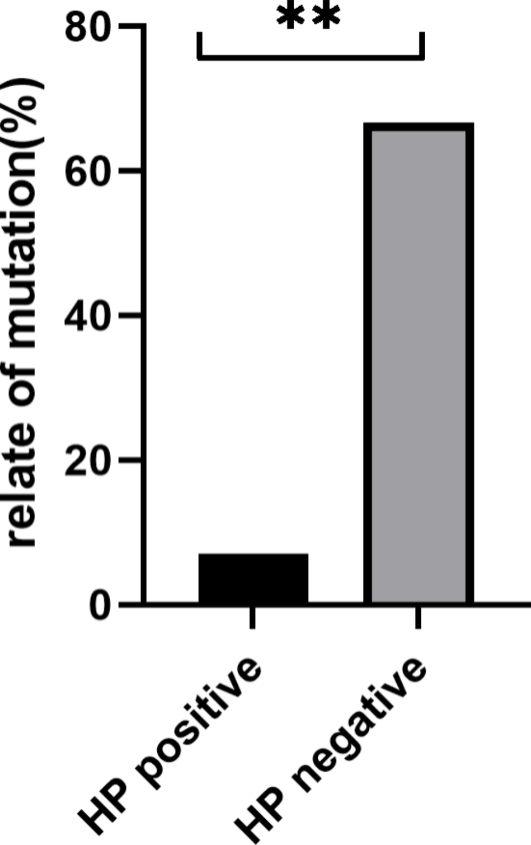
Mutation rate of ARID2 gene mutation in Hp-positive and Hp-negative groups, *P*<0.05

To clarify the relationship between gene mutations and clinicopathology, we performed a correlation analysis of the 28 genes with a mutation rate greater than 5% and the prognosis of the patients. The results indicate that mutations in *MTOR* are inversely related to the prognosis of patients (correlation coefficient = -0.45 and *P* = 0.011). Patients with *MTOR* gene mutation were more prone to relapse and metastasis relative to those without the mutation (Table 2). The gastric group was further divided into HP-positive(7.1%) and HP-negative groups (66.7%), with a large difference in the mutation rate of *ARID2* between the two groups; *P* = 0.002 ( Fig. 6).

**Table 2:** Correlation analysis of the 28 genes and the prognosis of the patients

Gene	Mutations or not	Follow-up /Outcome		*r*	*P*	Gene	Mutations or not	Follow-up /Outcome		*r*	*P*
		CR+PR+SD	PD+DEATH					CR+PR+SD	PD+DEATH		
ETV6	Yes	3(100.0)	0(0.0)	0.107	0.566	ITPKB	Yes	27(90.0)	3(10.0)	-0.06	0.749
	No	25(89.3)	3(10.7)				No	1(100.0)	0(0.0)		
NF1	Yes	4(100.0)	0(0.0)	0.126	0.499	EP300	Yes	15(88.2)	2(11.8)	-0.067	0.724
	No	24(88.9)	3(11.1)				No	12(92.3)	1(7.7)		
KLHL6	Yes	3(100.0)	0(0.0)	0.107	0.566	ARID2	Yes	9(90.0)	1(10.0)	-0.008	0.968
	No	25(89.3)	3(10.7)				No	19(90.5)	2(9.5)		
MYD88	Yes	2(66.7)	1(33.3)	-0.262	0.155	TNFAIP3	Yes	7(100.0)	0(0.0)	0.177	0.241
	No	26(92.9)	2(7.1)				No	21(87.5)	3(12.5)		
NOTCH1	Yes	2(66.7)	1(33.3)	-0.262	0.155	SETD2	Yes	5(83.3)	1(16.7)	-0.116	0.535
	No	26(92.9)	2(7.1)				No	23(92.0)	2(8.0)		
NOTCH2	Yes	3(100.0)	0(0.0)	0.107	0.566	MTOR	Yes	3(60.0)	2(40.0)	-0.45	0.011
	No	25(89.3)	3(10.7)				No	25(96.2)	1(3.8)		
PDGFRB	Yes	2(66.7)	1(33.3)	-0.262	0.155	KMT2C	Yes	8(80.0)	2(20.0)	-0.241	0.192
	No	26(92.9)	2(7.1)				No	20(95.2)	1(4.8)		
SPEN	Yes	2(66.7)	1(33.3)	0.107	0.566	ATM	Yes	21(91.3)	2(8.7)	0.056	0.764
	No	25(89.3)	3(10.7)				No	7(87.5)	1(12.5)		
TET2	Yes	3(100.0)	0(0.0)	0.107	0.566	CIITA	Yes	3(100.0)	0(0.0)	0.084	0.653
	No	25(89.3)	3(10.7)				No	25(89.3)	3(10.7)		
TNFRSF14	Yes	3(100.0)	0(0.0)	0.107	0.566	NIFKBIE	Yes	2(100.0)	0(0.0)	0.086	0.646
	No	25(89.3)	3(10.7)				No	26(89.8)	3(10.3)		
BCOR	Yes	2(100.0)	0(0.0)	0.086	0.646	ARID1A	Yes	24(88.9)	3(11.1)	-0.126	0.499
	No	26(89.8)	3(10.3)				No	4(100.0)	0(0.0)		
DNMT3A	Yes	2(100.0)	0(0.0)	0.086	0.646	KMT2A	Yes	22(95.7)	1(4.3)	0.306	0.094
	No	26(89.8)	3(10.3)				No	6(75.0)	2(25.0)		
FAS	Yes	2(100.0)	0(0.0)	0.086	0.646	BCORL1	Yes	22(95.7)	1(4.3)	0.306	0.094
	No	26(89.8)	3(10.3)				No	6(75.0)	2(25.0)		
JAK3	Yes	2(100.0)	0(0.0)	0.086	0.646	FAT1	Yes	10(90.9)	1(9.1)	0.015	0.937
	No	26(89.8)	3(10.3)				No	18(90.0)	2(10.0)		
KMT2D	Yes	19(100.0)	9(75.0)	0.412	0.021	APC	Yes	19(100)	9(75.0)	0.412	0.021
	No	0(0.0)	3(25.0)				No	0(0.0)	3(25.0)		

**Fig. 7:**
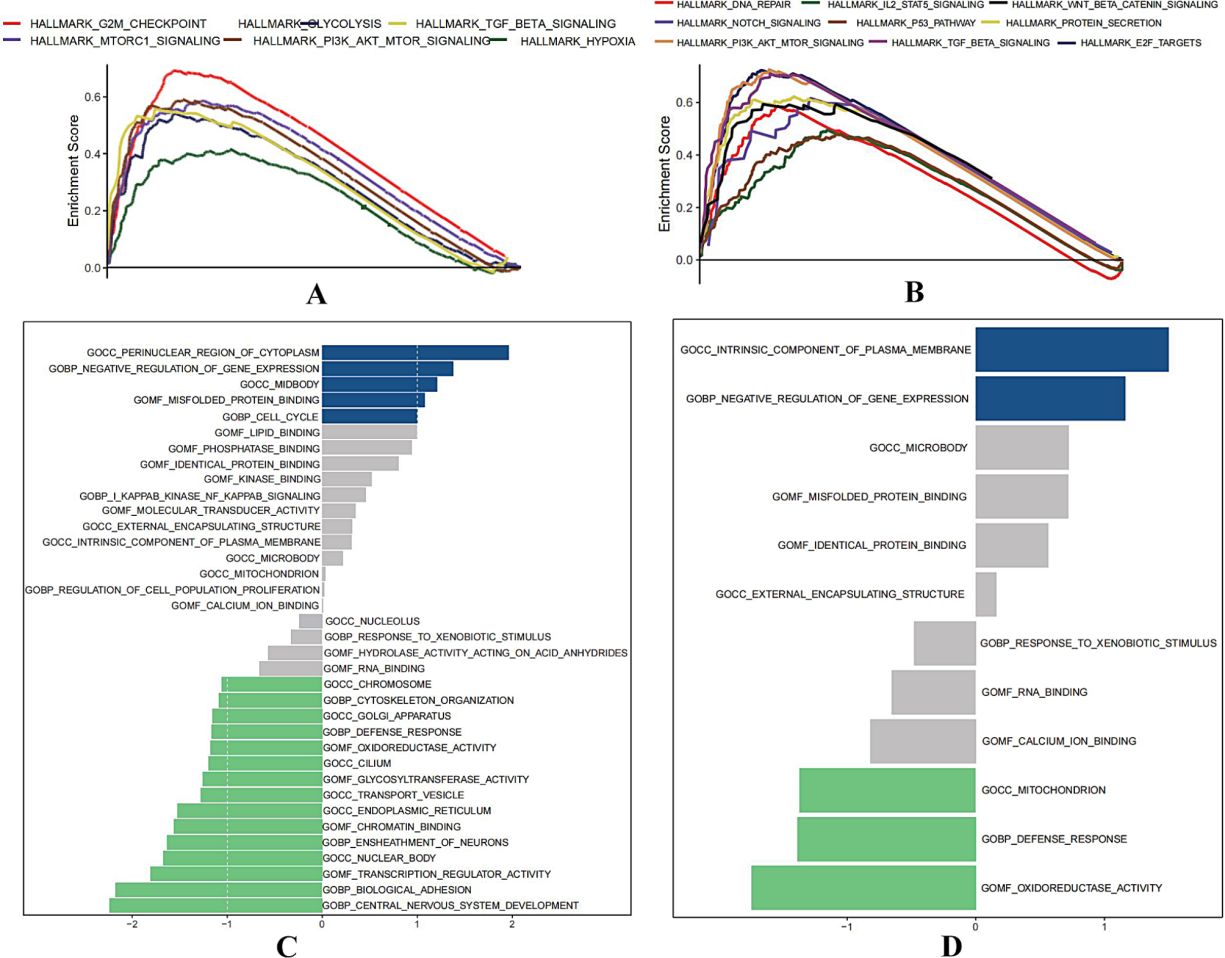
Enrichment of MTOR and ARID2 in lymphoma. **(A)** GSEA enrichment analysis were performed between the high and low MTOR group in lymphoma. **(B)**GSEA enrichment analysis were performed between the high and low ARID2 group in lymphoma. **(C)** GSVA enrichment analysis were performed between the high and low MTOR group in lymphoma.**(D)**GSVA enrichment analysis were performed between the high and low ARID2 group in lymphoma

In order to understand the signaling pathways and biological behaviors of these two genes(*MTOR*,* ARID2*)involved in the lymphoma database, we performed the pathway enrichment of the two genes through GSEA, and the results are presented in Fig. 7. Moreover, we found that the *MTOR* gene was mainly enriched in the PI3K/AKT/mTOR, *MTORC1*, and TGF-β, G2/M Checkpoint signaling pathway in the lymphoma database. In addition, it is also enriched in related pathways such as glycolysis and tissue hypoxia. However, the ARID2 gene was mainly enriched in the IL-2/STAT5, NOTCH, P53, PI3K/AKT/mTOR, TGF-β, and Wnt-β signaling pathways.

**Fig. 8:**
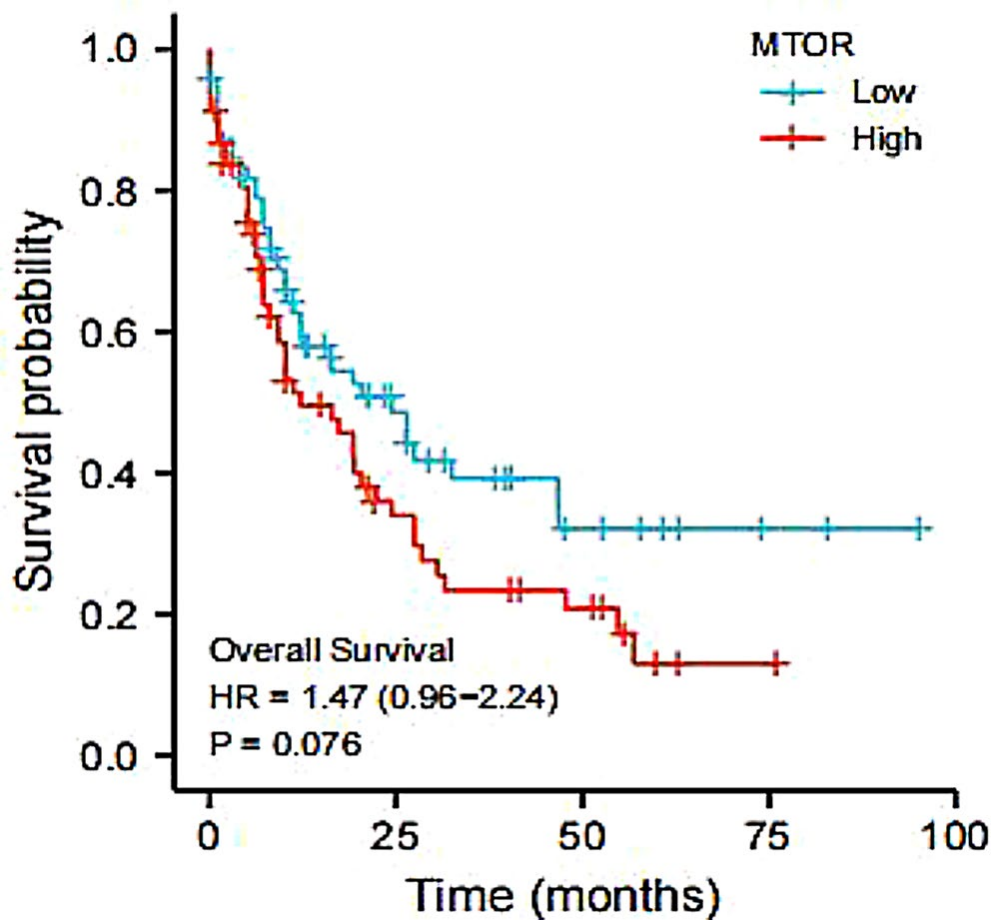
Survival analysis between high and low MTOR expression groups

The pathway enrichment of the two genes was performed through GSVA (Fig. 7C-D). In the cellular component (CC), the *MTOR* is mainly enriched in the perinuclear region of the cytoplasm, while the ARID2 is mainly enriched in the intrinsic component of the plasma membrane. In the biological process (BP)and molecular function (MF), the expression of both genes was observed to be identical. The MTOR and the ARID2 were mainly enriched in the negative regulation of gene expression and misfolded protein binding. The prognostic analysis of the *MTOR* gene showed a difference in survival curves between high and low expression groups.(Fig. 8).

## Discussion

MALT lymphoma was first described in 1983 by pathologists; Peter Isaacson and Dennis Wright [6] and was formally defined in 2008 in WHO’s classification of lymphocytic malignancies as mucosa-associated lymphoid tissue extra-nodal marginal zone lymphoma, a distinct type of lymphoma that is significantly different from the other inert B-cell lymphomas [7], and mainly occurs in the gastrointestinal tract.

IGH Gene rearrangement was detected by polypropylene gel electrophoresis and capillary electrophoresis in 31 patients, and both methods showed high sensitivity and accuracy, with IgHA and IgκA being the most common among the eight loci.

In this study, targeted sequencing of 31 patients were performed by next-generation sequencing, and the target genes involved 101 hot spot genes in lymphoma. The panel was produced by Beijing Jingjun Medical Technology Co. We detected 50 mutations, of which 31 genes had a mutation rate greater than 5%. These 31 genes were involved in epigenetic regulation (*KMT2C, SETD2, EP300, KMT2D, TET2, DNMT3A, KMT2A*, etc.), tumor suppression (*FAT1, BCOR, BCORL1, ETV6, ARID1A, ATM, ARID2*) and signaling pathways (*NOTCH1, NOTCH2, TNFAIP3, NF1, TNFRSF14, MYD88, KLHL6, PDGFRB, SPEN, TET2, FAS, JAK3, NFKBIE, MTOR, RELN, CIITA, APC*). Among them, the most commonly mutated gene is *ITPKB* with a 97% mutation rate, followed by *KMT2C* (58%), FAT1 (48%), and *EP300* (45%). It is known that *ITPKB* is ubiquitously expressed lipid kinase which can phosphorylate 1,4,5-trisphosphatidylinositol, an intracellular messengerthat is produced from 4,5-bisphosphatidylinositol through phospholipase C [8, 9]. This gene has not shown such a high mutation rate in the previous studies carried out on lymphoma; hence, further studies are needed to confirm whether *ITPKB* mutations are responsible for the development of MALT lymphoma in the GI tract. Additionally, *EP300* mutations were also high and these mutations inhibit *H3K27* acetylation, leading to excessive repression of gene transcription and various B-cell signaling dysregulations [10, 11]. Moreover, some studies have also demonstrated that EP300 mutations are involved in the activation of the NOTCH signaling pathway by regulating the expression of *FBXW7* [12]. *KMT2C* (Lysine methyltransferase 2 C), a gene responsible for tumor suppression in various myeloid and epithelial cells, is associated with hematologic and solid tumors (e.g., head, neck, breast, esophageal, lung, endometrial, bladder, and brain cancers) and is also responsible for affecting the inhibition of cell growth [13–15]. Some studies have also confirmed a worse prognosis in non-specific peripheral T-cell lymphomas with mutations in the FAT1 gene [16]. However, our study did not investigate the clinicopathological significance of these genes.

We found that the prognosis was poorer and more prone to relapse and metastasis in the MTOR mutant group, and mutations in this gene can work as a biomarker of a poor prognosis in the alimentary tract MALT lymphoma. We retrieved the lymphoma-related data through the database and performed a single-gene prognostic analysis, the results showed that the survival curve between the high and low *MTOR* expression groups did not reach statistical significance, but there had been a trend of poor prognosis. Moreover, rapamycin’s (mTOR) mammalian target is a serine/threonine kinase that is responsible for regulating cell growth and metabolism, cell proliferation, cell motility, cell survival, protein synthesis, autophagy, and transcription. It can be activated directly through the PI3K/AKT pathway stimulation or indirectly through DEPTOR inactivation; however, activation of the PI3K/AKT/mTOR signaling pathway is the main driver of cancer cell growth, proliferation, survival, and chemoresistance [17–20]. Aberrant activation ofPI3K/AKT/mTOR signaling pathway has been demonstrated in primary central nervous system lymphoma PCNSL [21]. Furthermore, this.

study also found that *MTOR* was strongly associated with a poor prognosis of the disease. Currently, mTOR inhibitors are widely used in the clinic to target mutations in this gene, namely rapamycin-like inhibitors, often referred to as “rapalogs”, which inhibit the mutated mTORC1. It has an overall response rate of 38% alone [22] and 59% in combination with melphalan (including 19% CR), which is the best response rate observed for targeted therapy in MCL. Based on these data, temsirolimus was approved as an orphan drug in Europe for relapsed condyloma lymphoma. Temsirolimus has an ORR of 30–40% in other NHL (FL, SLL, and aggressive lymphoma) and HL. Moreover, it may also have good efficacy in GI MALT lymphoma, particularly against refractory MALT lymphoma, with specific conclusions, to be confirmed by further studies.

By using the bioinformatics analysis, it was observed that the *MTOR* gene was mainly enriched in the PI3K/AKT/mTOR, MTORC1, TGF-β, and G2/M Checkpoint signaling pathway in the lymphoma database. This gene is mainly enriched in the perinuclear region of the cytoplasm and has a role in the negative regulation of gene expression and misfolded protein binding. We found that the main signaling pathway of this gene is PI3K / AKT / mTOR, and the activation of this pathway is also the main driving force for poor prognosis in lymphoma. In addition to drug inhibition, TGF-signaling was found to inhibit lymphoma growth in a variety of lymphomas(natural killer/ T cell lymphoma, diffuse large B-cell lymphoma), and repeated inactivation occurred in these lymphomas [23–25].

In addition to this, we divided 23 patients in the gastric group into Hp-positive and Hp-negative groups, and the mutation rate of the *ARID2* gene was significantly different between the two groups, among which the mutation of this gene was more often found in patients without Hp infection. It was known that Hp has a high infection rate in the population, and its causal relationship with chronic gastritis, peptic ulcer, and gastric cancer has been largely clarified. Further research, especially the finding that eradication of Hp can cause regression of gastric MALT lymphoma attracted more attention, and the relationship between Hp infection and gastric MALT lymphoma is recognized to be closer [26]. However, what are the causative factors in MALT lymphoma without Hp infection? Therapeutic challenges also persist, and according to our research, mutations are considered potentially important factors in pathogenesis. *ARID2* encodes a component of the SWI/SNF chromatin remodeling complex and a member of the AT-rich interaction domain (ARID) family of DNA-binding proteins involved in a variety of biological processes, including transcriptional regulation [27], cell cycle regulation [28, 29], embryonic development [30], and DNA damage repair [31]. *ARID2* mutations were first recognized in hepatocellular carcinoma (HCC), followed by colorectal cancer [32, 33]. To date, *ARID2* mutations have been reported in many human cancers, including melanoma, uroepithelial carcinoma, gastric adenocarcinoma, non-small cell lung cancer, and HCC [34]. Several studies have confirmed the tumor suppressive properties of *ARID2* [33, 35, 36]. Recently, shRNA-mediated *ARID2* knockdown in different colorectal cancer cell lines was demonstrated in a study by Bala et al. to cause significant alterations in the transcription levels of cancer-related target genes. More importantly, *ARID2* knockdown significantly enhanced tumor formation in nude mice, in addition to promoting a variety of tumorigenic features, including cell viability, proliferation, the ability to overcome contact growth inhibition and migration, and proposed *ARID2* as a novel tumor suppressor in colorectal cancer [37]. Moreover, this gene has not been widely studied in hematologic tumors. Research carried out by Bluemn et al. [38]demonstrated that deletion of the *ARID2* gene impaired the differentiation ability of hematopoietic stem cells and that this effect may be mediated through up-regulation of inflammatory pathways. Furthermore, Yamamoto et al. [39] identified a role of *ARID2* in multiple myeloma, the expression of this molecule, which may be associated with the prognosis of the disease and may be an effective therapeutic target for patients with lenalidomide-resistant multiple myeloma. In the study of myelodysplastic syndromes, H Sakai et al. [40] initially demonstrated that deletion of the *ARID2* gene may be a novel genetic and morphological phenotype of MDS. However, in our study, the high mutation rate of the *ARID2* gene in Hp-negative gastric MALT lymphoma had to draw our attention to the possibility that mutations in this gene may have a crucial role in Hp-negative gastric MALT lymphoma pathogenesis. Through the GSVA and GSEA analyses, we found that the main pathways enriched for *ARID2* were the IL-2/STAT5, NOTCH, P53, PI3K / AKT / mTOR, TGF-β, and Wnt-βsignaling pathways. Therefore, we can infer that the activation of these signaling pathways may be the major driving force of Hp-negative gastric MALT lymphoma.

In conclusion, mutated genes in GI MALT lymphoma frequently affect epigenetic regulation, tumor suppression, signaling pathways such as (BCR/NF-κB, notch), etc. Moreover, mutated genes in Hp-negative gastric MALT lymphoma frequently affect chromatin remodeling (*ARID2*), and we concluded that *ARID2* may have a critical role in Hp-negative gastric MALT lymphoma’s development. The spectrum of genetic lesions in lymphomas from different anatomical sites is not very different. mutations in *MTOR* may be a valid target for poor prognosis.

### Electronic supplementary material

Below is the link to the electronic supplementary material.

Supplementary Material 1

Supplementary Material 2

Supplementary Material 3

Supplementary Material 4

Supplementary Material 5

Supplementary Material 6

## Data Availability

No datasets were generated or analysed during the current study.

## Abbreviations

CR: Complete response
PR: Partial response
SD: Stable disease
PD: Progressive disease
