# Supplementary figures and images for: Revealing the clinical impact of MTOR and ARID2 gene mutations on MALT lymphoma of the alimentary canal using targeted sequencing
Source: Diagn Pathol. 2024 Jul 25;19:102. doi: 10.1186/s13000-024-01525-x (PMC11270975; doi:10.1186/s13000-024-01525-x)

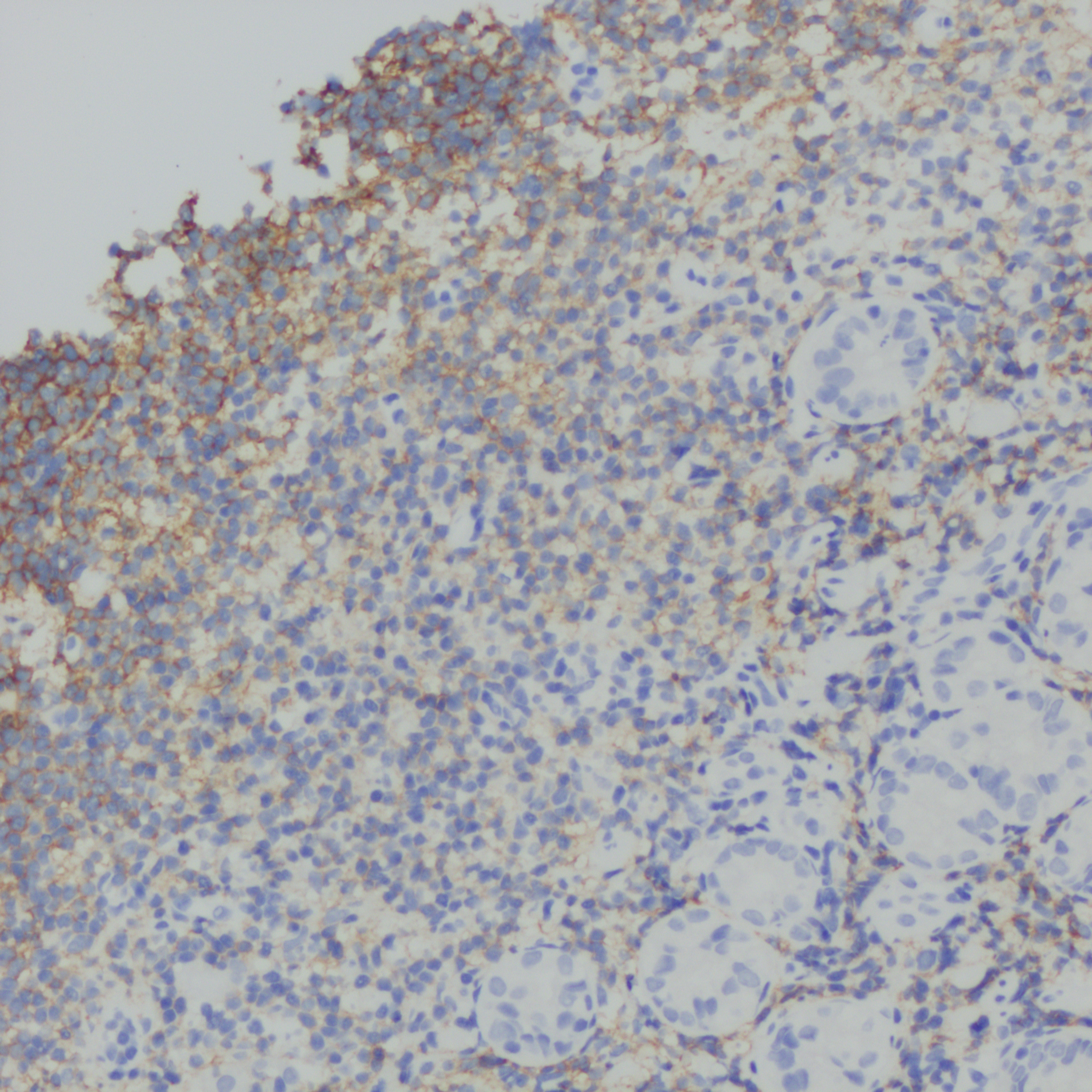

Supplement: Supplementary file 1 — Supplementary Material 1 [file 13000_2024_1525_MOESM1_ESM.jpg]

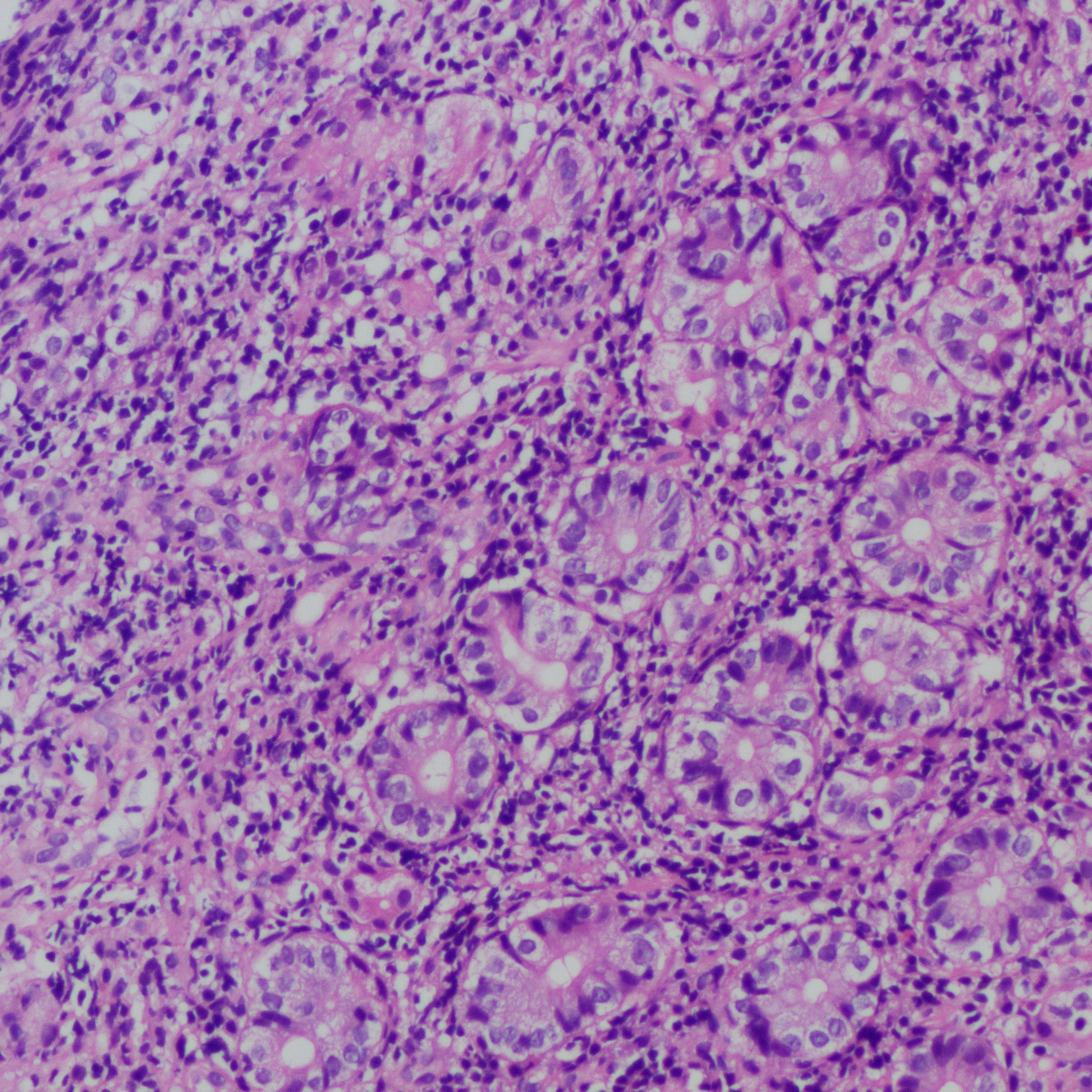

Supplement: Supplementary file 2 — Supplementary Material 2 [file 13000_2024_1525_MOESM2_ESM.jpg]

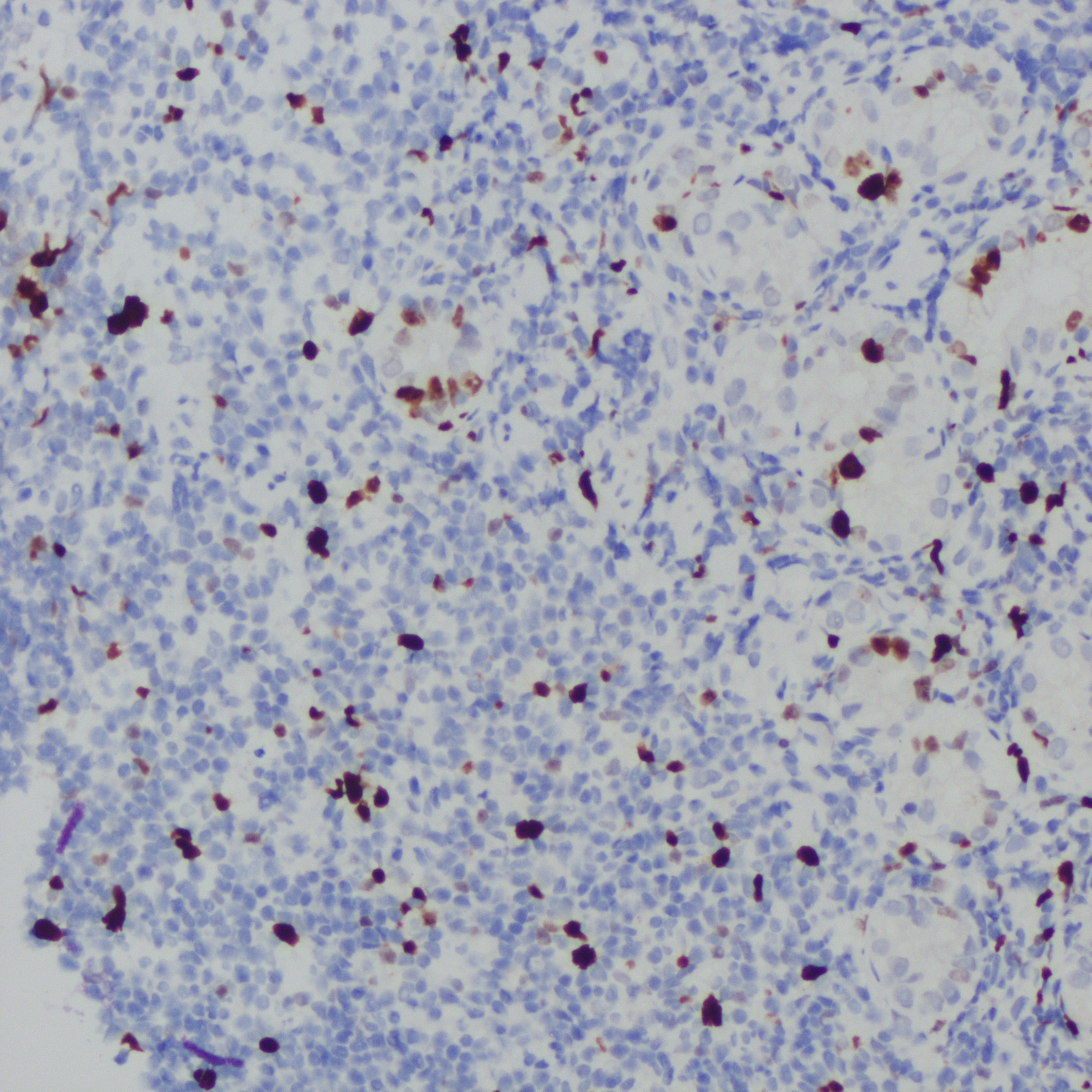

Supplement: Supplementary file 3 — Supplementary Material 3 [file 13000_2024_1525_MOESM3_ESM.jpg]

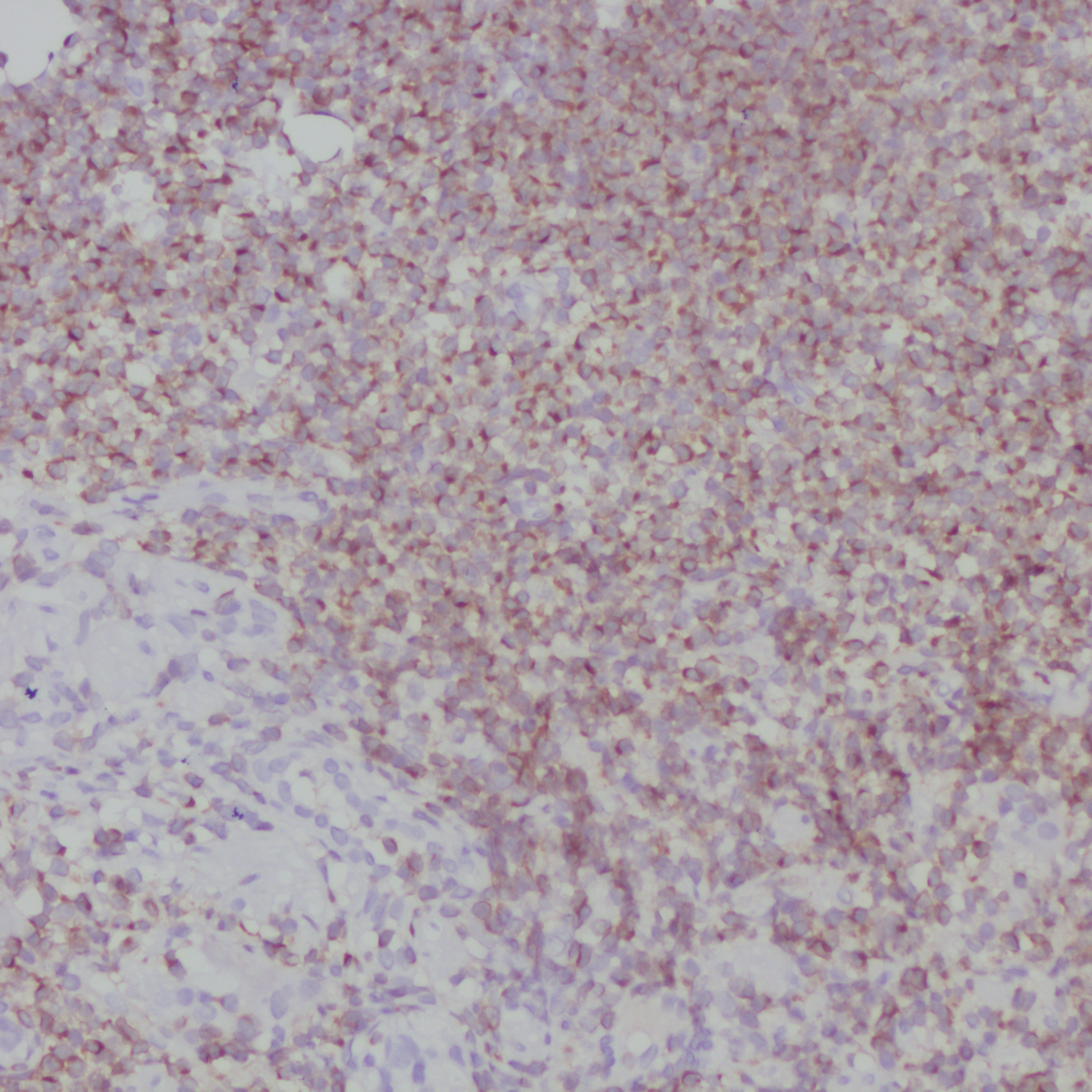

Supplement: Supplementary file 4 — Supplementary Material 4 [file 13000_2024_1525_MOESM4_ESM.jpg]

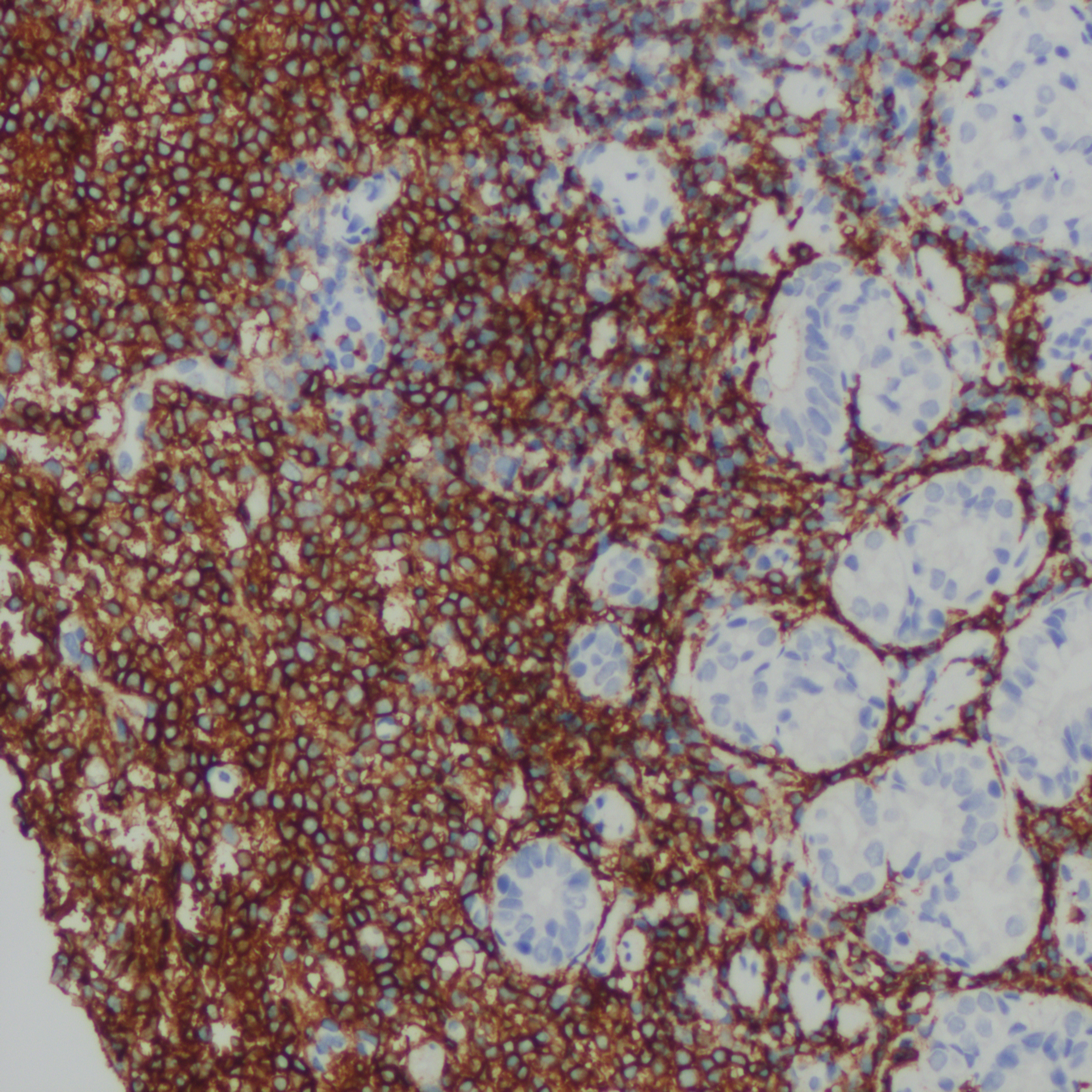

Supplement: Supplementary file 5 — Supplementary Material 5 [file 13000_2024_1525_MOESM5_ESM.jpg]
