# Supplementary material for: Revealing the clinical impact of MTOR and ARID2 gene mutations on MALT lymphoma of the alimentary canal using targeted sequencing
Source: Diagn Pathol. 2024 Jul 25;19:102. doi: 10.1186/s13000-024-01525-x (PMC11270975; doi:10.1186/s13000-024-01525-x)
Supplement: Supplementary file 6 — Supplementary Material 6 [file 13000_2024_1525_MOESM6_ESM.rtf]

Name of the main experimental reagent	Manufacturer (Place of origin)	
VAHTS®AmpSeqLibrary Prep Kit 
V3NA210 library building kit	Vazyme Biotech Co.,Ltd
	
TIANamP FFPE DNA Kit	TIANGEN BIOTECH (BEIJING)Co.,LTD.	
Ion Library TaqMan®Quantition Kit	Thermo Fisher Scientific Technology Co., Ltd	
AMPure XP nucleic acid purification kit	Beckman Coulter Life Sciences	
IGH Gene Clonality Assay for ABI Fluorescence Detection	Invivoscribe, Inc.	
IGK Gene Clonality Assay for ABI Fluorescence Detection	Invivoscribe, Inc.	
IGL Gene Clonality Assay for ABI Fluorescence Detection	Invivoscribe, Inc	
IGH+IGK clonal test kit
9-100-0011	Beijing Siercheng Biotechnology Co., LTD	
Rabbit anti-human Ki-67 monoclonal antibody	Fuzhou Maixin company	
Maxvision secondary antibody kit	Fuzhou Maixin company	
DAB substrate color developing reagent	Fuzhou Maixin company	
PBS	Fuzhou Maixin company	
Hematoxylin	Jabez Genetics LTD	
